# Data on amputation free survival of patients with lower limb peripheral artery disease classified according TASC II classification and a new crural index

**DOI:** 10.1016/j.dib.2016.05.039

**Published:** 2016-05-24

**Authors:** Juho M. Jalkanen, Jan-Erik Wickström, Maarit Venermo, Harri H. Hakovirta

**Affiliations:** a Department of Vascular Surgery, Turku University Hospital and University of Turku, Finland; b Department of Vascular Surgery, University Hospital of Helsinki, Finland

## Abstract

The results of amputation free survival (AFS) of a cohort of 887 caucasian patients is shown.

The data is based on further analyses of data presented in Jalkanen et al. (2016) [1]. The 36-month amputation free survival of patients divided in new crural vessel disease classification (Crural Index), aortoiliac TASC II classification, femoropopliteal TASC II classification and most severe segment is presented. Also, in depth demographic data is presented for each Crural Index group Jalkanen et al., 2016 [1].

## Specification Table

**Table:** 

Subject area	Medicine
More specific subject area	Epidemiology of peripheral arterial disease
Type of data	Tables, figures
How data was acquired	Retrospective analyses of patient files
Data format	Raw, analysed
Experimental factors	All cause survival, amputation free survival, TASC II classification and crural index were measured
Experimental features	Retrospective analyses of DSA images and 36-month patient survival and amputation free survival
Data source location	Turku University Hospital, Turku, Finland
Data accessibility	Data is with this article

## Value of the data

• This is the first analyses of correlation between AFS and crural index.
• The data demonstrates the challenging nature of extensive crural disease. The more extensive the atherosclerosis on crural vessels is, the more interventions are needed.
• Present data shows that in addition to poor survival and AFS, crural index IV is associated with conservative treatment and inability to treat.
• It also provides estimation of survival and amputation free survival for TASC II classification for aortoiliac and femoropopliteal segments [2], [3], [4].

## 1 Data

**Fig. 1:**
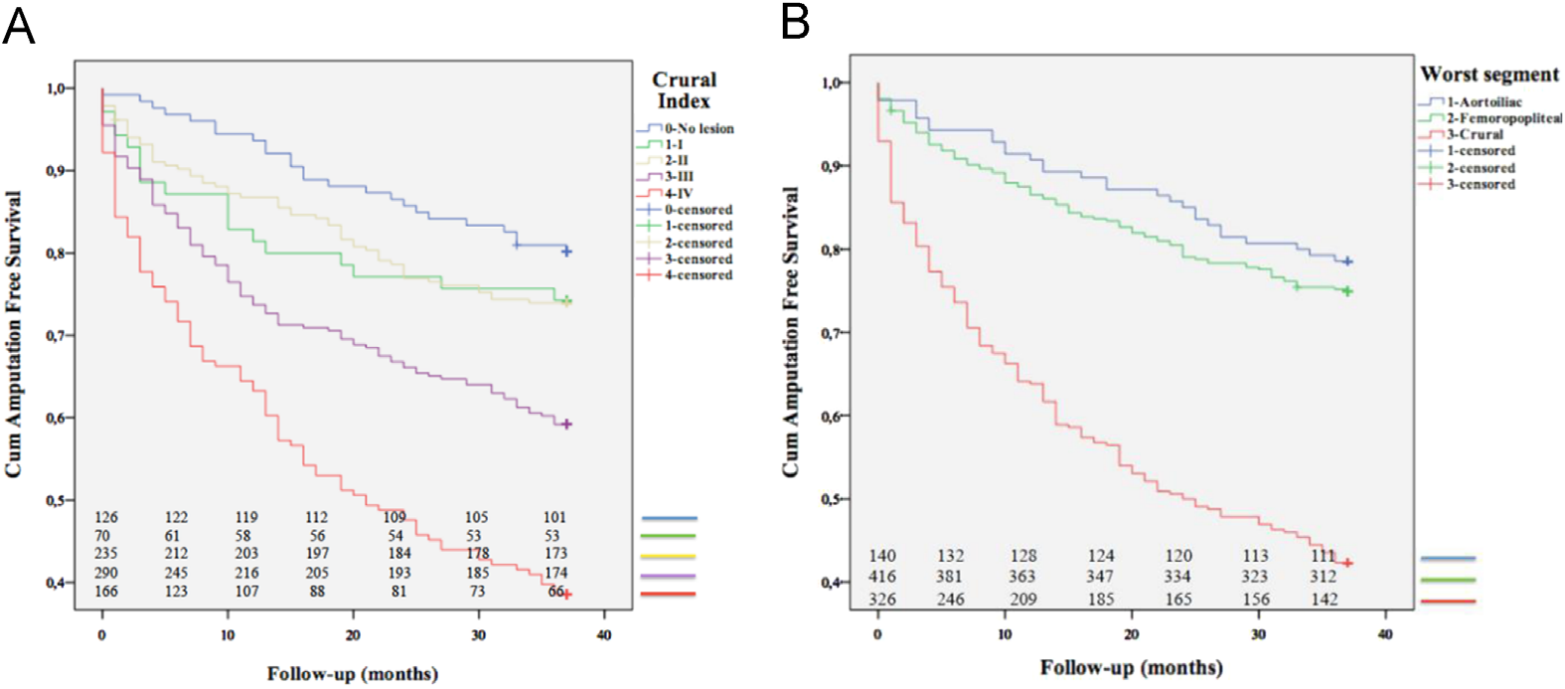
A**.** Kaplan-Meier curves show the cumulative amputation free survival (AFS) during 36-months follow-up. Separate curves for Crural Index I–IV and for patients with no detectable significant atherosclerotic lesion in crural arterial vessels. The survival curve of Crural Index IV demonstrates the poor prognosis of patients with extensive atherosclerosis in crural arteries. Numbers at risk for each curve marked at defined time-point. B**.** Kaplan–Meier curves demonstrating amputation free survival based on most severely diseased vascular segments. A more detailed presentation of data analysis is given in the methods section. The segments are marked as aorto-iliac (AI), femoro-popliteal (FP) and crural (Cr). Severe crural lesions result predict a poor AFS. Numbers at risk for each curve marked at defined time-point.

The presented data is acquired from analysis of amputation free survival and extent of atherosclerosis in crural vessels of PAD patients. Patient cohort was analysed according to widely used classification (TASC II) [2], [3], [4] and a new classification for the crural vessels [1]. The Kaplan-Meier curves for AFS are shown in Fig. 1A and B. Table 1A–E presents the mean AFS±SE for different classifications of arterial disease and disease level in lower limb arteries. Table 2A–E shows patient survival during 36-month follow-up divided correspondingly to Table 1 AFS Table 3.

## 2 Experimental design, materials and methods

The data is based on 887 consecutive patients admitted to the Department of Vascular Surgery at the Turku University Hospital (Turku, Finland) either for diagnostic DSA or for endovascular treatment of PAD from January 1st 2009 to July 30th 2011. All patients were included regardless of earlier PAD history. Deaths and amputations within the patient cohort were registered for the first 36-months, which was the cut-off point for follow-up.

### 2.1 DSA analysis

The index classification was as described in TASC II for aorto-iliac and femoro-popliteal segments. Aorto-iliac and femoro-popliteal segments TASC II classification A–D, (coded as 1–4) were for the statistical analyses. For the crural region, all three vessels were first analysed separately and a Crural Index was formed accordingly (see for further description [1]). In order to assess different vascular segments against each other, each patient was assigned into a specific group of disease localisation: 1) aorto-iliac, 2) femoro-popliteal or 3) crural, based on which 0–IV rating gave the highest number.

### 2.2 Statistical analyses

All statistical analyses were performed using the IBM SPSS version 22 statistics program. Continuous variables were expressed as mean±standard error (SE). Survival analyses were assessed by Kaplan–Meier curves and Log-rank statistics.

## Figures and Tables

**Table 1:** The analyses of treatments in each Crural index group. Unable to treat percentage of cases not being able to treat either for the technical reasons or patient unfit for demanded surgery. Conservative includes unable to treat and patients with claudication and requiring too extensive revascularisation procedures for clinical symptom. Endovascular procedures during 36-months follow-up to the initially worse leg. Surgical revascularisations to initially worse leg during 36-months follow-up. Treatments to the initially worse leg during 36-month follow-up, including both endovascular and surgical procedures. Amputation free survival (AFS) 1, 2 and 3 years.

A						
Crural Index	Grade 0		Grade I	Grade II	Grade III	Grade IV

Unable to treat	3.20%		11%	4.30%	5.20%	9.60%
Conservative	7.1%		20%	16%	18%	31%
Endovascular	54%		67%	65%	62%	49%
Surgery	49%		23%	30%	39%	32%
Treatments (mean±SE)	1.3±0.086		1.06±1.102	1.10±0.047	1.31±0.068	0.098±0.073
AFS 1, 2, 3 years	87%, 81%, 79%		77%, 76%, 73%	79%, 74%, 74%	67%, 60%, 58%	49%, 40%, 37%


B			
Most severe segment	Aorto-iliac	Femoro-popliteal	Crural

Unable to treat	2.10%	0.10%	14%
Conservative	7%	12%	31%
Endovascular	65%	61%	56%
Surgery	43%	44%	21%
Treatments (mean±SE)	1.28±0.072	1.36±0.052	0.89±0.045
AFS 1, 2, 3 years	86%, 79%, 78%	80%, 75%, 74%	51%, 44%, 41%

**Table 2:** Mean estimated amputation free survival during 36-months follow-up, SE and 95% CI presented in the table for A) Aorto-iliac (AI), B) Femoro-popliteal (FP), C) Crural (Cr) grades I–IV, D) Localization of significant atherosclerotic lesion, E) The most severe atherosclerotic segment. Log-rank test shown on the left row of the table. Number of patients at risk for each group *n*.

		(*n*)	Mean months±SE	95% CI; Lower−Upper Bound

A	AI I	92	32.1±1.06	30.1−34.2
	AI II	57	31.2±1.58	28.1−34.3
	AIII	34	27.6±2.27	23.1−32.0
*P*=0.010	AIIV	65	28.5±1.59	25.4−31.6
				
B	FP I	82	28.6±1.60	25.5±29.4
	FP II	140	29.2±1.09	27.0±31.3
	FP III	114	28.2±1.22	25.8±30.6
*p*=0.335	FP IV	329	27.3±0.758	25.8±28.8
				
C	Cr I	70	30.4±1.50	27.5−33.4
	Cr III	235	30.7±0.772	29.2−32.2
	Cr III	289	26.7±0.835	25.1−28.4
*P*<0.001	Cr IV	166	21.0±1.17	18.7−23.3
				
D	AI	25	36.5±0.47	35.6−37.4
	FP	61	32.7±1.30	30.2−35.3
	Cr	144	24.3±1.24	21.9−26.8
	AI+FP	36	32.6±1.48	29.7−35.5
	AI+Cr	48	33.0±1.45	30.2−35.8
	FP+Cr	428	27.0±0.685	25.7−28.4
*P*<0.001	AI+FP+Cr	138	27.7±1.14	25.4−29.9
				
E	AI	140	32.5±0.849	30.8−34.1
	FP	417	31.2±0.565	30.0−32.3
*P*<0.001	Cr	325	21.8±0.844	20.2−23.5
	Overall		27.9±0.460	27.0−28.8


**Table 3:** Mean estimated survival during 36-months follow-up, SE and 95% CI presented in the table for A) Aorto-iliac (AI), B) Femoro-popliteal (FP), C) Crural (Cr) grades I–IV, D) Localization of significant atherosclerotic lesion, E) The most severe atherosclerotic segment. Log-rank test shown on the left row of the table. Number of patients at risk for each group *n*.

		(n)	Mean Months±SE	95% CI; Lower-Upper Bound

A	AI I	(92)	32.4±1.01	30.5−34.4
	AI II	(57)	31.5±1.52	28.5−34.5
	AI III	(34)	27.6±2.31	23.0−32.1
*P*=0.128	AI IV	(66)	31.0±1.36	28.4−33.7
				
B	FP I	(82)	29.7±1.52	26.7−32.7
	FP II	(140)	29.9±1.05	27.8−31.9
	FP III	(114)	28.9±1.19	26.5−31.2
*P*=0.247	FP IV	(330)	28.8±0.700	27.4−30.2
				
C	Cr I	(70)	31.4±1.38	28.7−34.1
	Cr II	(235)	31.4±0.710	30.0−32.8
	Cr III	(289)	28.4±0.789	26.8−29.9
*P*=0.000	Cr IV	(167)	23.2±1.14	20.9−25.4
				
D	AI	(25)	36.2±0.48	35.6−37.5
	FP	(61)	32.8±1.26	30.3−35.3
	Cr	(144)	26.6±1.15	24.4−28.9
	AI+FP	(36)	33.3±1.43	30.5−36.1
	AI+Cr	(48)	33.3±1.40	30.5−36.0
	FP+Cr	(429)	28.4±0.648	27.1−29.6
*P*=0.030	AI+FP+Cr	(139)	28.9±1.06	26.8−31.0
				
E	AI	(141)	33.7±0.713	32.3−35.1
	FP	(416)	31.9±0.530	30.8−32.9
*P*=0.000	Cr	(325)	23.8±0.813	22.2−25.3
	Overall		29.2±0.430	28.3−30.0

^a^ Estimation is limited to the largest survival time 37 months				
